# 1023. Host Gene Expression Biomarkers to Distinguish Between Causes of Acute Respiratory Symptoms in Lung Transplant Recipients

**DOI:** 10.1093/ofid/ofab466.1217

**Published:** 2021-12-04

**Authors:** Julie M Steinbrink, Yiling Liu, Alice Gray, Omar Mohamedaly, Brittany Zick, Micah T McClain

**Affiliations:** 1 Duke University, Durham, North Carolina; 2 University of Colorado, Denver, Colorado; 3 Precision BioSciences, Durham, North Carolina

## Abstract

**Background:**

Long-term immunosuppression after lung transplantation increases susceptibility to a variety of respiratory infections that are often difficult to diagnose. Host gene expression patterns in circulating leukocytes may provide additional diagnostic information in these settings.

**Methods:**

107 lung transplant recipients (79% with cystic fibrosis) were enrolled at Duke University Medical Center over a 2-year period – 59% with acute respiratory symptoms, the remainder as healthy controls. Whole blood was collected by PAXGene for RNA sequencing. Prior to undergoing biomarker analysis, each case was adjudicated to the appropriate clinical phenotype: bacterial infection, viral infection, allograft rejection, and healthy. Logistic regression models were applied to gene expression data to identify classifiers capable of identifying each etiology.

**Results:**

In lung transplant recipients, 117 genes were upregulated at least 2-fold in the presence of viral infection compared to healthy transplant controls. These genes clustered into expected antiviral pathways, including type I interferon signaling, interferon gamma mediated signaling, and defense response to virus, although the magnitude of gene expression was significantly less than that seen in non-transplant cohorts.

Similar results were seen during bacterial infection (defense response to bacterium, antibacterial humoral response) and rejection (upregulation in defensins DEFA3 and DEFA4). Interestingly, despite the presence of immunosuppression, a previously published gene expression signature of respiratory infection (derived from non-immunosuppressed subjects) was able to differentiate between bacterial and viral infection with 100% accuracy.

**Conclusion:**

Even in the presence of systemic immunosuppression and regardless of presence/absence of cystic fibrosis, core canonical components of the host response to infection and rejection are seen. Gene expression signatures based on these conserved components offer the potential for diagnostic capability in the setting of nonspecific respiratory illness in these vulnerable hosts.

**Disclosures:**

**Julie M. Steinbrink, MD**, **CareDx** (Research Grant or Support) **Alice Gray, MD**, **CareDx** (Advisor or Review Panel member, Research Grant or Support, Speaker’s Bureau)**Polarean** (Advisor or Review Panel member)

